# Primary tuberculous osteomyelitis of the mandible mimicking a parotid fistula

**DOI:** 10.1590/S1808-86942011000300023

**Published:** 2015-10-19

**Authors:** Smita Upadhyay, Arpit Sharma, Vidisha Tuljapurkar, Jyoti P Dabholkar

**Affiliations:** 1 MS (resident); 2 MS (lecturer); 3 MBBS (resident); 4 MS (Professor and head); Department of ENT First Floor, OPD Building. Seth G.S. Medical College, Parel Mumbai -12 Maharashtra, India

**Keywords:** mandible, osteomyelitis, tuberculosis

## INTRODUCTION

Tuberculous osteomyelitis of the mandible is a rare entity; however the resurgence in the incidence of tuberculosis due to coinfection with HIV has brought back the focus on this age old disease^1^. Oral tuberculosis is usually secondary to pulmonary involvement however the systemic manifestations may be absent and oral lesion may be the presenting finding.

## CASE REPORT

A young girl of 14 years was referred to us with a progressively increasing right cheek swelling since 2 months. A similar swelling 3 months back was diagnosed as parotid abscess and drained. 10 days later she started complaining of a discharge from the same site. Patient remembered having undergone a tooth extraction 8 months back.

**Figure 1:**
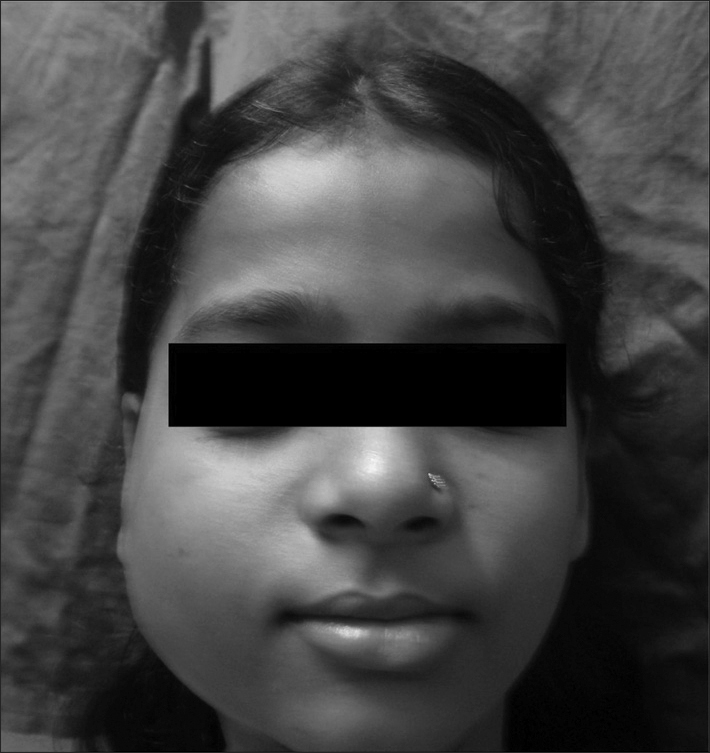
Clinical picture of the patient showing the right sided cheek swelling.

Examination revealed a firm swelling of about 5X5 present in the right parotid region reaching up to the angle of mandible. A discharging sinus was also present. (Fig. 1)

A plain radiograph of the chest was normal and FNAC from the swelling was inconclusive. Patient tested negative for HIV. Ultrasonography showed osteomyelitis of the mandible with a well defined abscess in the vicinity which was confirmed on CT. The outer cortex of the mandible at the angle and the neck showed a lytic destruction.

Patient was taken up for open biopsy. Intraoperatively the outer cortex of the mandible was destroyed. Caseous material was present and the sinus which was probed and found reaching up to the mandible was excised. As the parotid gland was normal, it was left behind.

The histopathology showed epitheloid cell granulomas composed of central area of caseous necrosis surrounded by epitheloid cells, Langhan type of giant cells and peripheral mantle of lymphocytes. A diagnosis of tuberculous osteomyelitis was thus made.

Patient was started on anti tubercular treatment. Patient completed her treatment with complete resolution of the swelling.

## DISCUSSION

Tuberculous osteomyelitis of the mandible is a rare entity. However in a country like India where tuberculosis is endemic, it should be an important differential when considering jaw swellings of long duration.

The disease most commonly involves young children. Mandible is rarely involved due to the less amount of cancellous bone^2^. The chief routes of spread are from infected sputum, surrounding soft tissue and haematogenous route. Wound following extraction of a tooth is also a possible conduit. The angle and the ramus of mandible are commonly involved. Destruction and erosion of the cortex occurs which is replaced by soft granulation tissue. Caseation occurs resulting in softening, liquefaction and formation of a subperiosteal abscess which can either burst intraorally or extraorally^3^. Sinuses which continue to discharge despite repeated course of antibiotics could also be the presenting symptom^4^.

Blurring of the bony details is the first sign and later a radiolucent area can also be seen on CT due to decalcification. The diagnosis is difficult in children because in its primary form it is paucibacillary and bacteriological correlation is always lacking. Absence of AFB in smears showing an otherwise characteristic cytological picture should not weigh against the diagnosis of TB^5^. Considering the overall prevalence of TB in India, the presence of epitheloid cell granuloma is indicative of TB unless proven otherwise^6^.

The treatment if initiated early results in complete resolution of the lesion. According to RNTCP the treatment advocated is of 6 months comprising of an intensive therapy of 2 months with Isoniazid, Rifampicin and Pyrazinamide and a maintenance therapy of 4 months with Rifampicin and Isoniazid.

## CONCLUSION

Primary tuberculous osteomyelitis is a rare entity. Increasing incidence of TB and HIV coinfection with emergence of drug resistance pose a major challenge for developing countries. Early diagnosis and prompt initiation of treatment is the only means to decrease the morbidity due to the disease.
